# Genome-wide association and linkage analyses of hemostatic factors and hematological phenotypes in the Framingham Heart Study

**DOI:** 10.1186/1471-2350-8-S1-S12

**Published:** 2007-09-19

**Authors:** Qiong Yang, Sekar Kathiresan, Jing-Ping Lin, Geoffrey H Tofler, Christopher J O'Donnell

**Affiliations:** 1 The National Heart, Lung and Blood Institute's Framingham Heart Study, Framingham, MA, USA; 2 Department of Biostatistics, Boston University School of Public Health, Boston, MA, USA; 3 Broad Institute of Harvard University and Massachusetts Institute of Technology, Cambridge, MA, USA; 4 Office of Biostatistics Research, NHLBI, National Institute of Health; Bethesda, MD, USA; 5 Royal North Shore Hospital Sydney, Australia; 6 Cardiology Division, Massachusetts General Hospital, Harvard Medical School, Boston, MA, USA

## Abstract

**Background:**

Increased circulating levels of hemostatic factors as well as anemia have been associated with increased risk of cardiovascular disease (CVD). Known associations between hemostatic factors and sequence variants at genes encoding these factors explain only a small proportion of total phenotypic variation. We sought to confirm known putative loci and identify novel loci that may influence either trait in genome-wide association and linkage analyses using the Affymetrix GeneChip 100K single nucleotide polymorphism (SNP) set.

**Methods:**

Plasma levels of circulating hemostatic factors (fibrinogen, factor VII, plasminogen activator inhibitor-1, von Willebrand factor, tissue plasminogen activator, D-dimer) and hematological phenotypes (platelet aggregation, viscosity, hemoglobin, red blood cell count, mean corpuscular volume, mean corpuscular hemoglobin concentration) were obtained in approximately 1000 Framingham Heart Study (FHS) participants from 310 families. Population-based association analyses using the generalized estimating equations (GEE), family-based association test (FBAT), and multipoint variance components linkage analyses were performed on the multivariable adjusted residuals of hemostatic and hematological phenotypes.

**Results:**

In association analysis, the lowest GEE p-value for hemostatic factors was p = 4.5*10^-16 ^for factor VII at SNP rs561241, a variant located near the *F7 *gene and in complete linkage disequilibrium (LD) (r^2 ^= 1) with the Arg353Gln *F7 *SNP previously shown to account for 9% of total phenotypic variance. The lowest GEE p-value for hematological phenotypes was 7*10^-8 ^at SNP rs2412522 on chromosome 4 for mean corpuscular hemoglobin concentration. We presented top 25 most significant GEE results with p-values in the range of 10^-6 ^to 10^-5 ^for hemostatic or hematological phenotypes. In relating 100K SNPs to known candidate genes, we identified two SNPs (rs1582055, rs4897475) in erythrocyte membrane protein band 4.1-like 2 (*EPB41L2*) associated with hematological phenotypes (GEE p < 10^-3^). In linkage analyses, the highest linkage LOD score for hemostatic factors was 3.3 for factor VII on chromosome 10 around 15 Mb, and for hematological phenotypes, LOD 3.4 for hemoglobin on chromosome 4 around 55 Mb. All GEE and FBAT association and variance components linkage results can be found at http://www.ncbi.nlm.nih.gov/projects/gap/cgi-bin/study.cgi?id=phs000007

**Conclusion:**

Using genome-wide association methodology, we have successfully identified a SNP in complete LD with a sequence variant previously shown to be strongly associated with factor VII, providing proof of principle for this approach. Further study of additional strongly associated SNPs and linked regions may identify novel variants that influence the inter-individual variability in hemostatic factors and hematological phenotypes.

## Background

The relationship of hemostasis and thrombosis with atherothrombotic cardiovascular disease has been extensively studied in the past decades. Elevated circulating levels of hemostatic factors, such as fibrinogen [1-3], plasminogen activator inhibitor (PAI-1) [4,5], von Willebrand factor (vWF) [6], tissue plasminogen activator (tPA) [4,5,7], factor VII (FVII) [8], and D-dimer [9,10] are linked to the development of atherothrombosis and are risk markers for coronary heart disease (CHD), stroke and other cardiovascular disease (CVD) events.

In addition to coagulation proteins, the cellular and rheological components of circulating blood have been implicated in CHD, stroke and peripheral arterial disease, including hematological phenotypes such as hematocrit (HCT), hemoglobin (Hgb), red blood cell count (RBCC) and size, mean corpuscular volume (MCV) and mean corpuscular hemoglobin (MCH) [11,12], as well as measures of platelet aggregation (induced by adenosine 5'-diphosphate (ADP), epinephrine (Epi) and collagen respectively) [12,13], and viscosity [14,15].

*Cis*-acting sequence variants in the following genes – fibrinogen-β (*FGB*), fibrinogen-α (*FGA*), fibrinogen-γ (*FGG*), FVII (*F7*), and PAI-1 (*SERPINE1*) – have been associated with corresponding levels of circulating hemostatic factor. By comprehensively characterizing common genetic variation at each of these loci, we have recently clarified that *cis*-acting variants, in sum, explain a modest proportion of phenotypic variation, ranging from 1% – 10% [16,17]. For hematological variables such as hematocrit and hemoglobin, sequence variation in the major hemoglobin genes is well described to be associated with anemias, such as beta- and alpha-thalassemia, and sickle cell anemia [18-20].

Systematic searches for novel genes beyond the known genetic determinants influencing these phenotypes have been carried out using genome-wide linkage analyses with microsatellite markers: Chromosome regions that may harbor novel loci influencing fibrinogen, PAI-1 [21,22], hematocrit, Hgb, RBCC, MCV and MCH [23,24], have been identified. However, linkage scans with microsatellite markers generally had low power to detect loci with small effects, and lacked precision in localizing the loci; thus, few novel loci have been identified.

The recent completion of a genome-wide scan using the Affymetrix GeneChip Human Mapping 100K single nucleotide polymorphism (SNP) set on participants in the Framingham Heart Study offered the opportunity to conduct a genome-wide association study (GWAS) and linkage scan for variants that influence hemostatic factors and hematological phenotypes.

## Methods

### Study participants and genotyping methods

The Framingham Heart Study design and the genotyping of the Affymetrix GeneChip Human Mapping 100K SNP set on Framingham Heart Study participants are detailed in the overview of this project [25]. To avoid potential bias due to genotyping artifacts, we limited the association analyses to 70987 SNPs on autosomes with minor allele frequency (MAF) ≥ 10%, genotyping call rate ≥ 80%, and Hardy-Weinberg equilibrium test p-value ≥ 0.001.

### Measurements of hemostatic factors and hematological phenotypes

Venous blood samples of Framingham Heart Study Offspring Cohort taken at the first and second examination cycles (1971–1975, and 1979–1983) were used to measure Hgb, RBCC, MCV and MCH, and samples taken at the fifth examination cycle (1991–1995) were used to measure all the hemostatic factors, platelet aggregation, D-dimer, and viscosity. Fibrinogen was additionally measured at the sixth (1995–1998) and seventh (1998–2001) examination cycles, and PAI-I antigen levels at the sixth exam. Details of the assessment of hemostatic factor levels have been described previously [17,26]. Plasma fibrinogen levels were measured using the Clauss method [27]. Plasma PAI-I antigen, tPA antigen, von Willebrand factor and FVII antigen were assessed using enzyme-linked immunosorbent assays.

The determination of hematological phenotypes has been detailed previously. Platelet aggregation was performed according to the method of Born [28]. The reagents used were epinephrine, ADP and collagen. The percent extent of aggregation in duplicate to epinephrine and ADP was determined in varying concentrations (0.01 to 15 mmol/L). For each subject, the aggregation response (yes/no) was also tested to a fixed concentration of arachidonic acid (5 mg/mL). The collagen lag time was measured in response to 1.9 mmol/L collagen. Participants who were taking aspirin were excluded from the analyses for platelet aggregation phenotypes as well as PAI-1 and tPA.

HCT was measured by the Wintrobe method [29]. Blood was collected and spun at 5000 rpm for 20 minutes in a balanced oxalate tube. The percent of total blood volume that was due to red blood cells was determined visually against a calibrated scale. MCV is the average volume of an individual's red blood cells determined as the ratio of HCT to RBCC. MCH is the average amount of hemoglobin of an individual's red cell determined as the ratio of Hgb to RBCC.

### Statistical methods

Standardized multivariable adjusted residuals of the hemostatic and hematological phenotypes were computed and used in all the linkage and association analyses. Covariates used in the adjustments were determined based upon what has been reported in the literature as potential risk factors for hemostatic factors or hematological phenotypes. Hardy-Weinberg equilibrium was examined using an exact chi-square test statistic [30]. Association between each SNP and each hemostatic or hematological phenotype was examined using a population based association method via generalized estimating equations (GEE) [31] and family-based association test (FBAT) [32], assuming an additive genetic model. Variance components linkage analyses were conducted using a subset of SNPs with pairwise r^2 ^< 0.5. Details of both association and linkage methods are described in the overview of this project [25].

In secondary analyses, we combined the GEE association tests results across multiple phenotypes that may share the common pathway to reduce the type I error rates, and possibly detect SNPs of smaller effect sizes. We ranked SNPs by the number of GEE test p-values less than 0.01, and then by the geometric mean of the GEE test p-values. We also examined the β coefficient from the GEE regression that is the change in the phenotype in one standardized deviation unit with an increment of a copy of the alphabetically second allele (for example, allele G for a SNP with alleles A and G). This analysis was conducted for a phenotype assessed using multiple measurement methods such as the platelet aggregation with ADP-, collagen-, and Epi-induced platelet aggregation; or for a phenotype with serial measurements such as fibrinogen level measured at examination cycles 5, 6 and 7.

We attempted to identify association of 100K SNPs in or within 60 kilo base pairs (kbp) of selected candidate genes previously reported to be associated with hemostatic factors or hematological phenotypes. For hemostatic factors and platelet aggregation phenotypes, we included the following candidate genes in the search: *F7*, fibrinogen gene cluster (*FGB, FGA, FGG*), *SERPINE1*, plasminogen activator-tissue (*PLAT*), *vWF *and integrin beta 3 (*ITGB3*). For hematological phenotypes excluding platelet aggregation, we included erythropoietin receptor *(EPOR)*, erythropoietin *(EPO)*, erythrocyte membrane protein band 4.1-like 2 (*EPB41L2*), Kruppel-like factor 1(*KLF1*), heme binding protein 2 (*HEBP2*), the hemoglobin gene clusters on chromosome 11: hemoglobin-β chain complex *(HBB)*, hemoglobin-δ *(HBD)*, hemoglobin-γ A *(HBG1)*, hemoglobin-γ G *(HBG2)*, hemoglobin-ε 1 (*HBE1*), and the hemoglobin gene clusters on chromosome 16: hemoglobin-α 1 (*HBA1*), hemoglobin-α 2 (*HBA2*), hemoglobin-μ (*HBM*).

## Results

Table 1 displays the hemostatic and hematological phenotypes analyzed in this study, as well as the number of individuals, examination cycles, and covariates used in multivariable models. The sample size ranged from 702 to 1073. Traits measured at multiple examinations were analyzed using multivariable adjusted residuals from each examination measure, and also the average of all the multivariable adjusted residuals from individual examination cycles.

**Table 1:** Description of hemostatic factors, hematological phenotypes, and covariates adjustment

Phenotype	Number of adjusted phenotypes	Offsrping cohort exam cycles†	Sample size (min-max)	Covariates adjusted
**Hemostatic factors:**				
fibrinogen	4	5, 6, 7 and average	986–1073	age (and its squared and cubic terms), sex, body mass index, prevalent cardiovascular disease, current cigarette smoking, hypertension treatment, systolic blood pressure, diastolic blood pressure, estrogen therapy (women only), alcohol intake, triglycerides, diabetes, total cholesterol, and the ratio of total cholesterol to high-density lipoprotein cholesterol.
PAI-1	3	5, 6 and average	788–1037	
tPA	1	5	786	
vWF	1	5	883	
FVII	1	5	886	
D-dimer	1	5	987	
				
**Hematological and rheological phenotypes**				
platelet aggregation (ADP-induced)	1	5	724	
platelet aggregation (collagen-induced)	1	5	702	
platelet aggregation (Epi-induced)	1	5	719	
Viscosity	1	5	832	
Hgb	3	1, 2, and average	903–1066	age, sex, height, weight, high-density lipoprotein cholesterol, total serum protein, alcohol intake, triglycerides, and current cigarette smoking
RBCC	3	1, 2, and average	903–1062	
MCH	3	1, 2, and average	903–1062	
Hematocrit*	3	1, 2, and average	903–1062	
MCV	3	1, 2, and average	903–1062	
WBC*	3	1, 2, and average	903–1062	

*Results for these two phenotypes are not reported in this manuscript. The complete results of all the phenotypes listed above can be found at http://www.ncbi.nlm.nih.gov/projects/gap/cgi-bin/study.cgi?id=phs000007

Among individuals who were included in the genotyping and had at least one hemostatic factor or platelet aggregation phenotype measured at examination cycle five, 52% were women, mean age was 52 years, and 6% had prevalent CVD. Among individuals who were included in the genotyping and had at least one hematological phenotype measured at examination cycle one or two, 52% were women, with a mean age over the two examinations of 36 years, and 2% had prevalent CVD.

### Association between SNPs and hemostatic and hematological phenotypes

We report the 25 SNPs with lowest GEE association test p-values in Table 2 for hemostatic factors, and in Table 3 for hematological phenotypes. The lowest GEE p-value (4.5*10^-16^) for hemostatic factors was obtained from the test of association between circulating levels of FVII and rs561241; this SNP resides near the *F7 *gene on chromosome 13 and is in complete linkage disequilibrium (LD) (r^2 ^= 1) with the Arg353Gln F7 SNP (rs6046) we previously reported to account for 9% of total phenotypic variance [16]. The lowest GEE p-value (6.9*10^-8^) for hematological phenotypes was obtained in the test of association between MCH and rs1397048 on chromosome 11 near the olfactory receptors, olfactory receptor, family 5, subfamily AP, member 2 (*OR5AP2*), olfactory receptor, family 5, subfamily AR, member 1 (*OR5AR1*), olfactory receptor, family 9, subfamily G, member 1(*OR9G1*) and olfactory receptor, family 9, subfamily G, member 4 (*OR9G4*). The 25 SNPs with lowest FBAT association test p-values are presented in Additional file 1, Table A1 and Table A2, respectively.

**Table 2:** The 25 SNPs with lowest GEE association test p-values with hemostatic factors measured at exam 5

Phenotype*	SNP	MAF	CHR	Physical Position (bp)^†^	**GEE p-val (Rank)**^††^	FBAT p-val^†††^	Genes within 60 kb
Fibrinogen	rs6683832	0.41	1	62,988,925	**5.6 × 10^-5^(4)**	3.4 × 10^-2^	*APG4C*
Fibrinogen	rs4861952	0.11	4	182,696,179	**3.5 × 10^-5^(24)**	8.2 × 10^-3^	
Fibrinogen	rs10506540	0.12	12	65,699,364	**2.2 × 10^-5^(16)**	3.3 × 10^-2^	
Fibrinogen	rs974673	0.15	14	33,550,265	**3.1 × 10^-5^(19)**	1.3 × 10^-3^	
FVII	rs966321	0.47	1	4,225,577	**7.6 × 10^-6^(6)**	4.5 × 10^-2^	
FVII	rs1245072	0.17	1	69,827,777	**1.8 × 10^-5^(13)**	7.5 × 10^-3^	*LRRC7*
FVII	rs2362932	0.24	1	242,240,325	**2.4 × 10^-5^(17)**	1.5 × 10^-2^	*SMYD3*
FVII	rs10496112	0.30	2	64,535,384	**3.3 × 10^-5^(23)**	7.4 × 10^-3^	*HSPC159*
FVII	rs1001254	0.13	3	7,909,333	**2.0 × 10^-5^(14)**	2.0 × 10^-2^	
FVII	rs4591494	0.19	3	7,957,827	**9.2 × 10^-6^(7)**	1.2 × 10^-4^	
FVII	rs10488360	0.30	7	4,184,450	**7.2 × 10^-6^(5)**	9.3 × 10^-3^	
FVII	rs727435	0.13	9	80,809,022	**9.6 × 10^-6^(8)**	2.8 × 10^-2^	
FVII	rs7938734	0.31	11	49,834,857	**3.2 × 10^-5^(21)**	7.0 × 10^-2^	
FVII	rs561241	0.12	13	112,808,035	**4.5 × 10^-16^(1)**	3.4 × 10^-4^	*MCF2L; AB116074; AK092739; AK123267; AB002360; F7; CR603372; F10; PROZ*
PAI1	rs10490733	0.14	2	137,872,229	**3.2 × 10^-5^(22)**	2.4 × 10^-2^	
PAI1	rs4460176	0.29	5	109,524,859	**3.1 × 10^-6^(3)**	7.6 × 10^-2^	
tPA	rs10493485	0.29	1	71,783,150	**1.6 × 10^-6^(2)**	1.1 × 10^-3^	*BC036771; NEGR1*
tPA	rs10494076	0.32	1	108,050,369	**3.1 × 10^-5^(20)**	2.3 × 10^-2^	*VAV3*
tPA	rs2029637	0.25	3	113,404,510	**1.3 × 10^-5^(10)**	6.1 × 10^-2^	*SLC9A10*
tPA	rs953377	0.28	6	23,998,670	**1.5 × 10^-5^(11)**	3.4 × 10^-2^	
vWF	rs9295740	0.14	6	27,797,481	**2.8 × 10^-5^(18)**	5.7 × 10^-3^	
vWF	rs202906	0.12	6	28,119,631	**2.1 × 10^-5^(15)**	1.1 × 10^-1^	*ZNF165*
vWF	rs708356	0.35	12	128,998,794	**1.5 × 10^-5^(12)**	2.4 × 10^-2^	*AK127723*
vWF	rs1954971	0.38	18	26,633,241	**1.0 × 10^-5^(9)**	1.6 × 10^-2^	
vWF	rs7319671	0.30	13	36,923,224	**3.6 × 10^-5^(25)**	1.1 × 10^-2^	

* All the phenotypes reported here were multivariable adjusted residuals from the measurements obtained at exam cycle 5.

^†^Physical position is in base pair (bp) and based on the May 2004 human reference sequence (NCBI Build 35).

^††^P-value from GEE genotype association test and rank of the GEE p-values in ascending order.

^†††^P-value from family-based association test using the FBAT program.

**Table 3:** The 25 SNPs with lowest GEE association tests p-values with hematological phenotypes

Phenotype*	SNP	MAF	CHR	Physical Position (bp)^†^	**GEE Pval (Rank)**^††^	FBAT Pval^†††^	Genes within 60 Kb
Hgb	rs4133289	0.19	1	156,267,010	**1.6 × 10^-7^(2)**	6.0 × 10^-4^	*OR10J1; OR10J5*
Hgb	rs1510107	0.41	2	53,071,788	**1.0 × 10^-5^(17)**	2.2 × 10^-3^	
Hgb	rs1160297	0.43	2	53,148,971	**1.0 × 10^-6^(4)**	8.3 × 10^-4^	
Hgb	rs2357013	0.45	2	53,177,780	**6.1 × 10^-6^(14)**	1.3 × 10^-3^	
Hgb	rs7844723	0.45	8	122,977,684	**2.1 × 10^-6^(5)**	2.6 × 10^-3^	
MCH	rs1829883	0.41	5	98,809,002	**5.8 × 10^-6^(11)**	1.4 × 10^-2^	
MCH	rs1200821	0.47	10	37,599,586	**5.9 × 10^-6^(13)**	1.0 × 10^-1^	*ANKRD30A*
MCH	rs1397048	0.40	11	56,222,675	**6.9 × 10^-8^(1)**	1.0 × 10^-2^	*OR5AP2; OR5AR1; OR9G1; OR9G4*
platelet aggregation (ADP-induced)	rs10493895	0.19	1	97,983,247	**1.6 × 10^-5^(23)**	4.5 × 10^-2^	*BC064027; DPYD*
platelet aggregation (ADP-induced)	rs10484128	0.15	14	97,712,325	**5.8 × 10^-6^(12)**	1.3 × 10^-3^	
platelet aggregation (collagen-induced)	rs848523	0.42	2	36,659,307	**2.1 × 10^-5^(24)**	4.3 × 10^-1^	*CRIM1*
platelet aggregation (collagen-induced)	rs565229	0.10	11	121,694,675	**3.7 × 10^-6^(6)**	2.6 × 10^-2^	
platelet aggregation (collagen-induced)	rs10506458	0.13	12	61,731,359	**4.5 × 10^-6^(9)**	1.1 × 10^-4^	
platelet aggregation (Epi-induced)	rs6811964	0.11	4	158,143,059	**1.3 × 10^-5^(20)**	5.5 × 10^-2^	*PDGFC*
platelet aggregation (Epi-induced)	rs1958208	0.32	14	81,194,793	**1.1 × 10^-5^(18)**	7.0 × 10^-1^	
platelet aggregation (Epi-induced)	rs10502583	0.17	18	28,011,131	**1.1 × 10^-5^(19)**	1.1 × 10^-2^	*RNF138; MEP1B*
RBCC	rs9253	0.18	1	37,627,906	**4.2 × 10^-6^(7)**	1.1 × 10^-5^	*FLJ11730; BC016328*
RBCC	rs10489087	0.13	4	13,470,685	**4.5 × 10^-6^(8)**	9.6 × 10^-4^	
RBCC	rs636864	0.21	6	149,567,299	**6.3 × 10^-6^(15)**	1.3 × 10^-4^	
RBCC	rs727979	0.14	6	149,635,613	**7.5 × 10^-6^(16)**	2.6 × 10^-5^	*MAP3K7IP2*
RBCC	rs6108011	0.28	20	7,500,504	**5.8 × 10^-6^(10)**	7.0 × 10^-2^	
Viscosity	rs10490258	0.11	2	40,717,605	**1.5 × 10^-5^(21)**	2.9 × 10^-2^	
Viscosity	rs1359339	0.18	6	18,606,507	**2.2 × 10^-5^(25)**	2.3 × 10^-2^	*IBRDC2*
Viscosity	rs10485968	0.20	7	81,108,037	**1.6 × 10^-5^(22)**	3.0 × 10^-3^	
Viscosity	rs7159841	0.21	14	46,933,585	**2.1 × 10^-7^(3)**	2.0 × 10^-1^	*MAMDC1*

* For Hgb, MCH and RBCC reported here, multivariable adjusted residuals from average of measurements over exam cycles 1 and 2 were used; for all platelet aggregation phenotypes, and viscosity reported here, multivariable adjusted residuals from measurements at examination cycle 5 were used.

^†^Physical position is in base pair (bp) and based on the May 2004 human reference sequence (NCBI Build 35).

^††^P-value from GEE genotype association test and rank of the GEE p-values in ascending order.

^†††^P-value from family-based association test using the FBAT program.

### Linkage results

Maximum multipoint LOD scores greater than 2 and the 1.5-LOD support intervals around the maximum LOD scores are presented in Table 4. The highest LOD score for hemostatic factors was 3.3 for factor VII at approximately 15 Mb on chromosome 10. The highest LOD for hematological phenotypes was 3.4 for Hgb at approximately 55 Mb on chromosome 4.

**Table 4:** Maximum LOD scores (≥2) on each chromosomes for hemostatic factors and hematological phenotypes

Phenotype*	SNP	CHR	Physical Position (bp)^†^	LOD	1.5 LOD Support Interval	
Fibrinogen	rs1273819	2	227,307,918	2.4	221,455,139	234,213,322
FVII	rs1542535	3	138,548,560	2.8	128,478,856	147,720,726
FVII	rs2400107	10	15,113,999	3.3	13,834,912	21,013,729
PAI1	143xd8	8	2,117,752	2.3	181,076	5,519,520
vWF	rs10514670	3	18,544,296	2.1	9,261,438	25,964,778
platelet aggregation (ADP-induced)	rs4148751	7	86,787,804	2.0	81,841,699	94,233,832
platelet aggregation (collagen-induced)	rs17310225	X	41,973,417	2.1	30,937,095	81,367,217
Hgb	rs2412522	4	54,779,471	3.4	41,552,790	57,899,713
Hgb	rs10514838	9	120,826,350	2.5	113,537,965	133,903,932
MCVavg12	rs38993	7	153,013,905	2.2	149,613,755	154,729,817
MCVavg12	rs9299952	11	19,075,037	3.3	12,241,666	21,560,867
MCVavg12	rs10522006	X	32,347,187	2.3	27,553,388	40,183,712
RBCC	rs956275	4	57,108,334	2.5	44,437,719	74,935,678
RBCC	rs2023048	6	162,441,307	2.9	156,253,205	165,513,097
RBCC	rs10500769	11	12,975,794	3.2	9,335,581	15,231,734
RBCC	rs10500770	11	13,053,050	3.2	9,335,581	15,231,734
RBCC	rs2178692	12	6,706,312	2.8	4,817,859	9,212,944
RBCC	rs10498633	14	91,996,705	2.6	74,270,406	93,717,052
RBCC	GATA27A03	15	100,152,332	2.3	98,566,644	100,152,332
RBCC	rs2271090	17	76,861,200	2.3	73,915,579	78,166,561
RBCC	rs486633	18	555,760	3.3	156,277	4,009,209
RBCC	rs20037	22	27,802,395	2.2	25,095,430	42,711,182

* For Hgb, MCH and RBCC reported here, multivariable adjusted residuals from average of measurements over exam cycles 1 and 2 were used; for all platelet aggregation phenotypes, fibrinogen, FVII, PAI1, vWF reported here, multivariable adjusted residuals from measurements at examination cycle 5 were used.

^†^Physical position is in base pair (bp) and based on the May 2004 human reference sequence (NCBI Build 35).

### Combining association tests across multiple phenotypes

The top 10 SNPs with most number of p-values < 0.01 and lowest mean p-values are reported in Tables 5 and 6 for platelet aggregation phenotypes and fibrinogen levels respectively. The top ranked SNP for platelet aggregation was rs10500631 on chromosome 11 located near an olfactory gene cluster. The p-values of the GEE association test for ADP-, collagen- and epinephrine-induced platelet aggregation levels with this SNP were all less than 0.01, with average p-value 0.007 over the three tests. The range of the regression coefficients was 0.19–0.24, indicating the effect size was consistently estimated across the three phenotypes.

**Table 5:** Top 10 ranked SNPs in combining GEE association tests of ADP-induced, Collagen-induced and Epi-induced platelet aggregation levels

									GEE P-value of individual phenotypes		
									
SNP	MAF	CHR	Physical Position (bp)*	Mean GEE p-value	Range of Beta Coefficient^† ^(min, max)		Number of Phenotypes with GEE P-value < 0.01	Gene within 60 Kb	ADP	Collagen	Epi
rs10500631	0.13	11	4,882,639	6.6 × 10^-3^	0.19	0.24	3	*OR51S1; OR51T1; OR51A7; OR51G2; OR51G1; OR51A4; OR51A2*	9.7 × 10^-3^	7.8 × 10^-3^	3.8 × 10^-3^
rs930323	0.18	1	221,172,525	1.9 × 10^-3^	-0.26	-0.16	2	*CNIH3*	3.8 × 10^-2^	1.6 × 10^-3^	1.2 × 10^-4^
rs7033457	0.25	9	7,383,934	2.6 × 10^-3^	0.15	0.24	2		2.5 × 10^-3^	3.7 × 10^-2^	2.0 × 10^-4^
rs2974490	0.11	5	113,433,775	2.7 × 10^-3^	0.07	0.28	2		4.2 × 10^-1^	8.7 × 10^-5^	5.2 × 10^-4^
rs10517543	0.13	4	40,332,115	3.2 × 10^-3^	0.13	0.27	2	*AF262323; FLJ20273*	5.8 × 10^-3^	1.0 × 10^-1^	5.2 × 10^-5^
rs2916601	0.13	5	89,556,637	3.5 × 10^-3^	0.11	0.29	2		9.7 × 10^-2^	3.2 × 10^-4^	1.4 × 10^-3^
rs4350297	0.18	10	106,463,038	3.9 × 10^-3^	-0.24	-0.10	2	*SORCS3*	3.5 × 10^-4^	1.0 × 10^-1^	1.7 × 10^-3^
rs7956194	0.42	12	108,340,545	4.3 × 10^-3^	0.09	0.20	2	*FLJ37587; KCTD10; BC040062; AK056912; BC051266; UBE3B*	3.6 × 10^-3^	1.1 × 10^-1^	2.0 × 10^-4^

* Physical position is in base pair (bp) and based on the May 2004 human reference sequence (NCBI Build 35).

^†^β coefficient is the change in the phenotype in one standardized deviation unit with an increment of a copy of the alphabetically higher allele.

**Table 6:** Top 10 ranked SNPs in combining GEE association tests of fibrinogen levels measured at examination cycles 5, 6 and 7

SNP	MAF	CHR	Physical Position (bp)*	mean GEE P-value	Range of β Coefficients^† ^(min, max)		Number of Phenotypes with GEE P-value < 0.01	Gene within 60 Kb	GEE P-value of individual phenotypes		
									**Exam 5**	**Exam 6**	**Exam 7**

rs4861952	0.11	4	182,696,179.00	4.9 × 10^-4^	-0.28	-0.17	3		3.5 × 10^-5^	9.2 × 10^-3^	3.6 × 10^-4^
rs1869733	0.15	8	63,970,954.00	5.5 × 10^-4^	-0.23	-0.16	3	*FLJ39630*	6.7 × 10^-5^	4.1 × 10^-3^	6.1 × 10^-4^
rs9317390	0.47	13	63,625,531.00	6.5 × 10^-4^	0.13	0.17	3		3.8 × 10^-3^	1.9 × 10^-4^	3.8 × 10^-4^
rs1561478	0.15	4	61,317,252.00	7.3 × 10^-4^	0.15	0.22	3		3.0 × 10^-4^	1.6 × 10^-4^	8.6 × 10^-3^
rs277330	0.16	5	53,489,351.00	1.1 × 10^-3^	-0.22	-0.16	3	*ARFRP2*	1.5 × 10^-3^	1.9 × 10^-4^	4.1 × 10^-3^
rs1649053	0.41	10	59,991,493.00	1.2 × 10^-3^	0.13	0.18	3		2.8 × 10^-3^	5.8 × 10^-3^	1.0 × 10^-4^
rs2175271	0.09	15	34,097,057.00	1.5 × 10^-3^	-0.26	-0.20	3		6.5 × 10^-3^	3.1 × 10^-4^	1.8 × 10^-3^
rs9323656	0.06	14	77,394,323.00	1.5 × 10^-3^	-0.31	-0.27	3	*ADCK1; AK096919*	1.2 × 10^-3^	2.5 × 10^-3^	1.3 × 10^-3^
rs1423741	0.42	16	59,874,592.00	1.8 × 10^-3^	-0.17	-0.11	3		7.6 × 10^-4^	9.4 × 10^-3^	7.6 × 10^-4^
rs10497881	0.32	2	205,818,470.00	2.2 × 10^-3^	0.14	0.17	3	*ALS2CR19*	4.8 × 10^-4^	5.0 × 10^-3^	4.5 × 10^-3^

* Physical position is in base pair (bp) and based on the May 2004 human reference sequence (NCBI Build 35).

^†^β coefficient is the change in the phenotype in one standardized deviation unit with an increment of a copy of the alphabetically higher allele.

For fibrinogen, the top ranked SNP was rs4861952 on chromosome 4, which was also listed in the Table 2 as one of the 25 most significantly associated SNPs with hemostatic factors. This SNP was consistently associated with fibrinogen levels across three examination cycles with effect size ranging from -0.28 to -0.17.

### Association of SNPs in known candidate genes

100K SNPs residing in or near known candidate genes for hemostatic factors are presented in Table 7. Among the candidate genes for hemostatic factors, no 100K SNP was in or within 60 kb of *PLAT*. Only SNPs in or near the rest of the candidate genes (*F7*, *FGG*, *FGA*, *FGB*, *ITGB3*, *SERPINE1 *and *vWF*) are presented. Among all these associations, three reached nominal significance (p-value < 0.05): rs561241 for factor VII, and rs6950982 and rs6956010 for PAI-1.

**Table 7:** Association between SNPs in/near known hemostatic candidate genes, and the corresponding phenotypes

Candidate Gene	SNP	CHR	Physical Position (bp)*	MAF	Position to Gene	Function	Phenotype^†^	GEE Association Pvalue
*F7*	rs561241	13	112,808,035	0.12	NEAR	unknown	FVII, exam 5	4.5 × 10^-16^
*FGG*	rs2066864	4	155,883,300	0.19	IN	intron	Fibrinogen, average	0.239
*FGG*	rs2066864	4	155,883,300	0.19	IN	intron	Fibrinogen, exam 5	0.711
*FGG*	rs2066864	4	155,883,300	0.19	IN	intron	Fibrinogen, exam 6	0.095
*FGG*	rs2066864	4	155,883,300	0.19	IN	intron	Fibrinogen, exam 7	0.586
*FGG*	rs1074801	4	155,933,889	0.49	NEAR	unknown	Fibrinogen, average	0.667
*FGG*	rs1074801	4	155,933,889	0.49	NEAR	unknown	Fibrinogen, exam 5	0.898
*FGG*	rs1074801	4	155,933,889	0.49	NEAR	unknown	Fibrinogen, exam 6	0.521
*FGG*	rs1074801	4	155,933,889	0.49	NEAR	unknown	Fibrinogen, exam 7	0.608
*ITGB3*	rs4525555	17	42,692,948	0.31	IN	intron	Platelet Aggregation, ADP, exam 5	0.231
*ITGB3*	rs4525555	17	42,692,948	0.31	IN	intron	Platelet Aggregation, Collagen, exam 5	0.375
*ITGB3*	rs4525555	17	42,692,948	0.31	IN	intron	Platelet Aggregation, epinephrine, exam 5	0.292
*ITGB3*	rs10514919	17	42,697,128	0.24	IN	intron	Platelet Aggregation, ADP, exam 5	0.495
*ITGB3*	rs10514919	17	42,697,128	0.24	IN	intron	Platelet Aggregation, Collagen, exam 5	0.331
*ITGB3*	rs10514919	17	42,697,128	0.24	IN	intron	Platelet Aggregation, epinephrine, exam 5	0.302
*ITGB3*	rs2015729	17	42,709,492	0.36	IN	intron	Platelet Aggregation, ADP, exam 5	0.326
*ITGB3*	rs2015729	17	42,709,492	0.36	IN	intron	Platelet Aggregation, Collagen, exam 5	0.919
*ITGB3*	rs2015729	17	42,709,492	0.36	IN	intron	Platelet Aggregation, epinephrine, exam 5	0.963
*SERPINE1*	rs6950982	7	100,360,038	0.18	NEAR	unknown	PAI1, average	0.187
*SERPINE1*	rs6950982	7	100,360,038	0.18	NEAR	unknown	PAI1, exam 5	0.035
*SERPINE1*	rs6950982	7	100,360,038	0.18	NEAR	unknown	PAI1, exam 6	0.688
*SERPINE1*	rs6956010	7	100,360,470	0.19	NEAR	unknown	PAI1, average	0.218
*SERPINE1*	rs6956010	7	100,360,470	0.19	NEAR	unknown	PAI1, exam 5	0.053
*SERPINE1*	rs6956010	7	100,360,470	0.19	NEAR	unknown	PAI1, exam 6	0.740
*VWF*	rs917858	12	5,952,199	0.32	IN	intron	wVW, exam 5	0.398
*VWF*	rs917859	12	5,952,374	0.32	IN	intron	wVW, exam 5	0.413
*VWF*	rs2239138	12	5,952,819	0.32	IN	intron	wVW, exam 5	0.487
*VWF*	rs216901	12	5,975,129	0.48	IN	intron	wVW, exam 5	0.297
*VWF*	rs216903	12	5,975,760	0.48	IN	intron	wVW, exam 5	0.316
*VWF*	rs216904	12	5,976,279	0.34	IN	intron	wVW, exam 5	0.584

* Physical position is in base pair (bp) and based on the May 2004 human reference sequence (NCBI Build 35).

^†^'NEAR' means within 60 kb to a gene; 'IN' means within a gene.

^††^Phenotypes are multivariate adjusted residuals.

Among the candidate genes for hematological traits, no 100K SNP was in or within 60 Kb of *EPOR*, *EPO*, *KLF1*, *HBA1*, *HBA2*, *HBM*. For the rest of the candidate genes, associations between hematological phenotypes and 100K SNPs in/near *EPB41L2*, the beta hemoglobin gene cluster on chromosome 11 (*HBB*, *HBD*, *HBG1*, *HBG2*, *HBE1*), and HEBP2 are presented in Table 8. The most significant associations were SNP rs1582055 near *EPB41L2 *with hematocrit (p = 7.7 × 10^-5^), Hgb (p = 2.9 × 10^-4^), and RBCC (p = 3.9 × 10^-4^); SNP rs4897475 with hematocrit (p = 1.6 × 10^-4^) and Hgb (p = 6.0 × 10^-4^).

**Table 8:** Association between hematological phenotypes and SNPs in/near known candidate genes

							GEE Association Test P-values^††^				
							
Candidate Gene	SNP	CHR	Physical Position (bp)*	MAF	Position to Gene^†^	Function	Hematocrit	Hgb	MCH	MCV	RBCC
*EPB41L2*	rs7741731	6	131,152,063	0.17	NEAR	unknown	0.488	0.326	0.847	0.549	0.163
*EPB41L2*	rs1582055	6	131,186,056	0.3	NEAR	unknown	7.7 × 10^-5^	2.9 × 10^-4^	0.427	0.762	3.9 × 10^-4^
*EPB41L2*	rs1413753	6	131,192,197	0.34	NEAR	intron	0.003	0.007	0.691	0.730	0.007
*EPB41L2*	rs1413754	6	131,192,319	0.3	NEAR	intron	0.242	0.158	0.561	0.039	0.016
*EPB41L2*	rs4075265	6	131,208,150	0.33	IN	untranslated	0.001	0.004	0.470	0.506	0.002
*EPB41L2*	rs915171	6	131,226,607	0.26	IN	intron	0.712	0.486	0.355	0.232	0.273
*EPB41L2*	rs9285468	6	131,233,823	0.33	IN	intron	0.003	0.005	0.437	0.921	0.022
*EPB41L2*	rs6938586	6	131,251,097	0.18	IN	intron	0.878	0.511	0.578	0.661	0.951
*EPB41L2*	rs3777442	6	131,251,481	0.48	IN	intron	0.017	0.124	0.834	0.568	0.044
*EPB41L2*	rs4897472	6	131,254,831	0.49	IN	intron	0.002	0.022	0.972	0.551	0.009
*EPB41L2*	rs4897475	6	131,274,643	0.28	IN	intron	1.6 × 10^-4^	6.0 × 10^-4^	0.633	0.485	0.011
*EPB41L2*	rs1334689	6	131,275,701	0.31	IN	intron	0.001	0.005	0.703	0.639	0.020
*EPB41L2*	rs7748468	6	131,297,742	0.3	IN	intron	0.002	0.005	0.616	0.956	0.009
*EPB41L2*	rs2105250	6	131,325,875	0.28	IN	unknown	0.013	0.002	0.050	0.696	0.076
*EPB41L2*	rs9321263	6	131,340,606	0.33	IN	unknown	0.461	0.137	0.188	0.095	0.056
*EPB41L2*	rs1811949	6	131,340,872	0.32	IN	unknown	0.511	0.210	0.176	0.119	0.076
*EPB41L2*	rs9321265	6	131,345,926	0.14	IN	unknown	0.985	0.909	0.734	0.941	0.896
*EPB41L2*	rs7761311	6	131,352,801	0.37	IN	unknown	0.986	0.553	0.202	0.066	0.143
*EPB41L2*	rs1412540	6	131,368,085	0.24	IN	unknown	0.006	0.006	0.356	0.725	0.008
*EPB41L2*	rs6907809	6	131,374,355	0.21	IN	unknown	0.739	0.928	0.153	0.049	0.281
*EPB41L2*	rs951251	6	131,432,905	0.15	NEAR	unknown	0.851	0.789	0.419	0.009	0.075
*HBB, HBD*	rs4128714	11	5,151,240	0.25	NEAR	unknown	0.557	0.402	0.345	0.175	0.431
*HBB, HBD, HBG1, HBG2, HBE1*	rs2105819	11	5,216,303	0.46	NEAR	unknown	0.007	0.039	0.128	0.027	0.463
*HBB, HBD, HBG1, HBG2, HBE1*	rs968856	11	5,217,152	0.47	NEAR	unknown	0.013	0.049	0.202	0.016	0.576
*HBB, HBD, HBG1, HBG2, HBE1*	rs10488676	11	5,225,373	0.48	NEAR	unknown	0.051	0.134	0.174	0.047	0.735
*HBB, HBD, HBG1, HBG2, HBE1*	rs10488675	11	5,254,606	0.27	NEAR	unknown	0.050	0.180	0.798	0.077	0.920
*HBG1, HBG2, HBE1*	rs3898916	11	5,284,996	0.38	NEAR	unknown	0.089	0.165	0.353	0.260	0.367
*HEBP2*	rs10499199	6	138,811,539	0.16	NEAR	intron	0.503	0.585	0.557	0.412	0.972
*HEBP2*	rs10499200	6	138,813,645	0.16	NEAR	intron	0.706	0.742	0.535	0.382	0.793
*HEBP2*	rs10499201	6	138,818,107	0.16	NEAR	intron	0.633	0.725	0.487	0.414	0.897
*HEBP2*	rs6902919	6	138,819,479	0.21	NEAR	intron	0.789	0.977	0.225	0.148	0.541
*HEBP2*	rs6926204	6	138,820,053	0.16	NEAR	intron	0.887	0.979	0.429	0.381	0.660

* Physical position is in base pair (bp) and based on the May 2004 human reference sequence (NCBI Build 35).

^†^'NEAR' means within 60 kb to a gene; 'IN' means within a gene.

^†† ^The p-values are from the GEE association test on multivariable adjusted phenotypes listed below.

## Discussion

We conducted a GWAS and a genome-wide linkage analyses for hemostatic factors and hematological phenotypes measured in Framingham Heart Study Offspring participants. We identified a highly significant association between factor VII level and SNP rs561241 in complete LD with the *F7 *SNP rs6046 (Arg353Gln) previously demonstrated to explain about 9% of total phenotypic variation. This association is significant after Bonferroni correction for multiple testing (we used a conservative α = 5 × 10^-8^), and confirms the strong association at this locus that has previously been reported by us and others. This SNP was also significant (p-value = 3.4 × 10^-4^) at a nominal α level 0.05 for FBAT and linkage test (LOD = 1.8, p-value = 0.002), but not after Bonferroni correction. That may be explained by the well known fact that FBAT and linkage test are less powerful than population-based association tests.

FBAT lacks power to detect variants that explain small proportion of variance for this study. It is difficult to distinguish true positives from false ones among FBAT results because it was evident that few 100K SNPs explain a large proportion of variance for hemostatic factors or hematological phenotypes. Given that there is no evidence for major population substructure in FHS [33] and there is greater power from use of GEE testing, we emphasize our population-based GEE analysis results in this report. Linkage analyses have the same problem of low power to detect small effects. However, a linkage peak can be caused by loci in linkage but not in LD with the SNPs, or by several loci of small effects in the region. Thus linkage peaks deserve additional attention. For example, we identified a linkage peak on chromosome 10 for multivariate adjusted factor VII. The SNP underneath the peak is rs2400107. However, the GEE association p-value was 0.52. This could occur because rs2400107 was linked but not in LD with the disease locus (loci) under the peak, or because this linkage peak was caused by several loci of small effects, or this peak was a false positive. Therefore, a more careful examination of the association results of SNPs under the linkage peak along with potentially additional genotyping may be needed to confirm the linkage results.

Among the SNPs with top GEE p-values in single phenotype or multiple phenotypes analyses, only a few resided near genes that were known for a likely role in hemostasis and thrombosis and hematological biology. For hemostatic factors, the *cis*-acting SNP rs561241 near *F7 *gene was associated with factor. For hematological phenotypes, we identified rs6811964 near *PDGFC*, platelet derived growth factor-C. It has been shown that PDGFC highly expressed in vascular smooth muscle cells, renal mesangial cells and platelets, and was likely involved in platelet biology [34]. This SNP was found associated with Epi-induced platelet aggregation (P = 10^-5^, Table 3), with ADP-induced platelet aggregation at nominal significance (P = 0.02), and with collagen-induced platelet aggregation at borderline nominal significance (P = 0.08). Other associations were found with SNPs in genes not clearly related to the phenotypes, or with SNPs that are not in known genes. These associations, together with other findings from this GWAS, must be viewed as hypotheses that warrant further testing in other cohorts.

Although we only summarized results for multivariable adjusted phenotype, we have also conducted linkage and association analyses for age-sex adjusted phenotypes. It is possible that the effects of some loci may be mediated through the covariates included in multivariable adjustment, and thus only associated with age and sex adjusted phenotypes. Among the 52 SNPs that were associated with age and sex adjusted hemostatic factors or hematological phenotypes with a GEE p-value equal or less than 10^-5^, 28 SNPs had a GEE p-value greater than 10^-5 ^with multivariable adjusted phenotypes. However, no age and sex adjusted GEE p-value for the 28 SNPs reached genome-wide significance (p-value < 5 × 10^-8^), and no new highly plausible candidate genes resided within 60 Kb of these SNPs. The full disclosure results of all analyses, including the age-sex adjusted analyses, can be viewed at http://www.ncbi.nlm.nih.gov/projects/gap/cgi-bin/study.cgi?id=phs000007.

There are some limitations to this study. The participants are Caucasian and thus the results may not be generalizable to other racial groups. The study sample size was relatively small, and as such, we may have insufficient power to detect small effects. To avoid worsening the multiple testing problem, we performed only sex-pooled and not sex-specific analyses. There may be some SNPs that are associated with some phenotypes only in female or male undetected in the current study. The advantages of this study are that we had family data, which enabled us to also apply family-based association tests that are robust to population admixture, and linkage analyses that can detect loci not in LD but in linkage with any 100K SNP. The study subjects were recruited without regarding to their phenotypic values, which makes the analyses of multiple phenotypes possible without the need to correct ascertainment bias.

Finally, compared with studies focused only on SNPs within candidate genes, GWAS approaches are unbiased and as such they have the advantage of detecting novel genes or confirming genes that are not well-known to have an influence on a phenotype. However, since the current GWAS uses only a subset of all the SNPs in HapMap [35], it may miss some genes due to lack of coverage. For the same reason, GWAS data usually are not enough to study a candidate gene comprehensively. To understand the roles played by each SNP in a candidate gene, additional genotyping, and single-SNP and haplotype analyses are needed. A large GWAS involving more than 550,000 SNPs in more than 9000 participants of FHS will be available for analysis later in 2007, providing increased power for detection of smaller effects for the hemostatic and hematological phenotypes.

## Conclusion

In summary, we have tested for association and linkage using the Affymetrix 100K SNPs and a set of hemostatic factor and hematological phenotypes. We have confirmed a previously reported association, providing proof of principle (a "positive control") for the GWAS approach. Our results provide a set of hypotheses that warrant testing in additional studies.

## Abbreviations

ADP = adenosine 5'-diphosphate; bp = base pair(s); CHD = coronary heart disease; CVD = cardiovascular disease; CHR = chromosome; EPB41L2 = erythrocyte membrane protein band 4.1-like 2; EPO = erythropoietin; EPOR = erythropoietin receptor; FBAT = family-based association test; FGB = fibrinogen-β; FGA = fibrinogen-α; FGG = fibrinogen-γ; FHS = Framingham Heart Study; FVII = factor VII; GAW = Genetic Analysis Workshop; GEE = generalized estimating equations; GWAS = genome-wide association study; HBA1 = hemoglobin-α 1; HBA2 = hemoglobin-α 2; HBB = hemoglobin-β chain complex; HBD = hemoglobin-δ; HBE1 = hemoglobin-ε 1; HBG1 = hemoglobin-γ A; HBG2 = hemoglobin-γ G; HBM = hemoglobin-μ; HCT = hematocrit; HEBP2 = heme binding protein 2; Hgb = hemoglobin; ITGB3 = integrin, beta 3 (platelet glycoprotein IIIa, antigen CD61); kb = kilo base pairs (=1,000 bp); KLF1 = Kruppel-like factor 1; LD = linkage disequilibrium; MAF = minor allele frequency; MCV = mean corpuscular volume; MCH = mean corpuscular hemoglobin; Mb = mega base pairs (=1,000,000 bp); OR5AP2 = olfactory receptor, family 5, subfamily AP, member 2; OR5AR1 = olfactory receptor, family 5, subfamily AR, member 1; OR9G1 = olfactory receptor, family 9, subfamily G, member 1; OR9G4 = olfactory receptor, family 9, subfamily G, member 4; PAI-1 = plasminogen activator inhibitor; PDGFC = platelet derived growth factor-C; RBCC = red blood cell count; SERPINE1 = serpin peptidase inhibitor, clade E, member 1; SNP = single nucleotide polymorphism; tPA = tissue plasminogen activator; vWF = von Willebrand factor; WBC = white blood cell.

## Competing interests

The authors declare that they have no competing interests.

## Authors' contributions

QY took a leading role in study design, results summarization and interpretation, and manuscript drafting. SK contributed to the study design, summarization and interpretation of the results, and the manuscript revision. JPL contributed to the study of hematological phenotypes. GHT contributed to the measurement and study of the hemostatic traits. COD contributed to study design, results summarization and manuscript drafting and revision. All authors read and approved the final manuscript.

## Supplementary Material

Additional file 1 The 25 SNPs with lowest FBAT association test p-values are presented in Additional data file 1, Table A1 and Table A2, respectively. Click here for file
