# Supplementary material for: Genome-wide association and linkage analyses of hemostatic factors and hematological phenotypes in the Framingham Heart Study
Source: BMC Med Genet. 2007 Sep 19;8(Suppl 1):S12. doi: 10.1186/1471-2350-8-S1-S12 (PMC1995619; doi:10.1186/1471-2350-8-S1-S12)
Supplement: Additional file 1 — The 25 SNPs with lowest FBAT association test p-values are presented in Additional data file 1, Table A1 and Table A2, respectively. [file 1471-2350-8-S1-S12-S1.doc]

# Additional data files

| **Table A1. The 25 SNPs with lowest FBAT association test p-values with hemostatic factors measured at exam 5.** | | | | | | | |  | |
| --- | --- | --- | --- | --- | --- | --- | --- | --- | --- |
| Phenotype* | SNP | MAF | CHR | Physical Position (bp) ^†^ | GEE Pval^††^ | **FBAT Pval**  **(Rank)** ^†††^ | Genes within 60kb |  | |
| Fibrinogen | rs1516391 | 0.33 | 3 | 2,697,320 | 2.3X10^-1^ | **4.8X10^-5^(8)** | *CNTN4* |  | |
| Fibrinogen | rs1527323 | 0.49 | 3 | 74,939,582 | 1.4X10^-4^ | **5.5X10^-5^(10)** |  |  | |
| Fibrinogen | rs10503657 | 0.16 | 8 | 19,086,599 | 1.4X10^-2^ | **7.9X10^-5^(14)** |  |  | |
| Fibrinogen | rs7083579 | 0.15 | 10 | 6,560,590 | 3.1X10^-2^ | **1.1X10^-4^(23)** | *AY702977;PRKCQ* |  | |
| Fibrinogen | rs10502708 | 0.20 | 18 | 34,676,501 | 1.2X10^-1^ | **4.4X10^-5^(7)** |  |  | |
| FVII | rs2554297 | 0.26 | 1 | 60,152,206 | 5.8X10^-3^ | **9.7X10^-5^(22)** | *CYP2J2;AK126061;C1orf87;BC027878* |  | |
| FVII | rs1674067 | 0.11 | 6 | 6,284,376 | 8.2X10^-4^ | **8.8X10^-5^(17)** | *F13A1;FLJ33708* |  | |
| FVII | rs1055252 | 0.25 | 13 | 25,516,922 | 3.1X10^-3^ | **6.0X10^-5^(11)** | *TMEM46* |  | |
| FVII | rs1209912 | 0.32 | 21 | 39,071,512 | 4.3X10^-1^ | **1.2X10^-4^(25)** | *C21orf24;ETS2* |  | |
| PAI1 | rs719575 | 0.37 | 4 | 26,755,119 | 6.8X10^-2^ | **7.2X10^-5^(12)** | *STIM2* |  | |
| PAI1 | rs8543 | 0.12 | 5 | 172,523,943 | 1.3X10^-3^ | **1.6X10^-5^(1)** | *BNIP1* |  | |
| PAI1 | rs10508393 | 0.17 | 10 | 9,657,021 | 2.8X10^-3^ | **7.4X10^-5^(13)** |  |  | |
| PAI1 | rs10500777 | 0.17 | 11 | 13,232,887 | 3.6X10^-1^ | **9.1X10^-5^(18)** | *ARNTL* |  | |
| PAI1 | rs1064125 | 0.20 | 12 | 1,471,239 | 2.1X10^-2^ | **9.2X10^-5^(19)** | *RAB6IP2* |  | |
| PAI1 | rs10506728 | 0.16 | 12 | 75,999,549 | 3.3X10^-2^ | **8.4X10^-5^(16)** | *E2F7* |  | |
| PAI1 | rs8061107 | 0.44 | 16 | 56,859,330 | 2.3X10^-3^ | **1.1X10^-4^(24)** | *HSPC065* |  | |
| tPA | rs9328354 | 0.36 | 6 | 6,289,006 | 4.5X10^-1^ | **9.3X10^-5^(21)** | *F13A1;FLJ33708* |  | |
| tPA | rs2108179 | 0.43 | 7 | 41,746,584 | 6.2X10^-4^ | **3.7X10^-5^(4)** | *GLI3* |  | |
| tPA | rs10498413 | 0.46 | 14 | 48,204,040 | 7.8X10^-3^ | **3.3X10^-5^(3)** |  |  | |
| tPA | rs8092348 | 0.13 | 18 | 35,919,395 | 2.4X10^-2^ | **9.2X10^-5^(20)** |  |  | |
| vWF | rs10498257 | 0.31 | 2 | 231,818,543 | 6.4X10^-2^ | **2.0X10^-5^(2)** | *PSMD1* |  | |
| vWF | rs6837060 | 0.23 | 4 | 36,109,680 | 1.5X10^-2^ | **8.0X10^-5^(15)** | *CENTD1;BC030111* |  | |
| vWF | rs1010242 | 0.40 | 10 | 16,011,241 | 4.1X10^-3^ | **4.8X10^-5^(9)** |  |  | |
| vWF | rs7979865 | 0.28 | 12 | 22,759,432 | 5.9X10^-3^ | **4.3X10^-5^(6)** | *ETNK1* |  | |
| vWF | rs10483836 | 0.26 | 14 | 71,071,367 | 1.1X10^-3^ | **3.7X10^-5^(5)** | *SIPA1L1* |  | |
|  |  |  |  |  |  |  |  |  | |
|  |  |  |  |  |  |  |  |  | |
| * All the phenotype reported here were multivariable adjusted residuals from the measurements obtained at exam cycle 5.  ^†^ Physical position is in base pair (bp) and based on the May 2004 human reference sequence (NCBI Build 35).  ^††^ P-value from GEE genotype association test. | | | | | | | |  |  |
| ^†††^ P-value from family-based association test using the FBAT program, and rank of FBAT p-values in ascending order. | | | | | | | |  | |

Table A2. The 25 SNPs with lowest FBAT association test p-values with hematological factors.

| phenotype* | SNP | MAF | CHR | Physical Position (bp) ^†^ | gee pval^††^ | **fbat pval**  **(Rank)** ^†††^ | Genes within 60kb |  |
| --- | --- | --- | --- | --- | --- | --- | --- | --- |
| Hgb | rs10518194 | 0.17 | 4 | 79440669 | 1.3X10^-3^ | **1.8X10^-5^(7)** | *FRAS1* | |
| Hgb | rs10483957 | 0.50 | 14 | 81899972 | 3.4X10^-1^ | **9.0X10^-5^(22)** |  | |
| Hgb | rs6575266 | 0.29 | 14 | 92099147 | 4.4X10^-3^ | **7.4X10^-5^(18)** | *RIN3;BC070062* | |
| MCH | rs584747 | 0.12 | 6 | 49,875,549 | 1.3X10^-2^ | **1.8X10^-5^(8)** | *CRISP3;PGK2;CRISP1* | |
| MCH | rs2217711 | 0.16 | 9 | 8,858,177 | 1.3X10^-3^ | **9.1X10^-5^(24)** | *PTPRD* | |
| MCH | rs10509327 | 0.15 | 10 | 72,264,168 | 2.0X10^-3^ | **8.0X10^-5^(20)** | *SGPL1* | |
| MCH | rs1408617 | 0.41 | 13 | 47,326,516 | 9.4X10^-2^ | **7.2X10^-5^(17)** |  | |
| platelet aggregation (ADP-induced) | rs218428 | 0.17 | 1 | 37,213,316 | 1.2X10^-2^ | **4.8X10^-5^(14)** | *GRIK3;AJ249210* | |
| platelet aggregation (ADP-induced) | rs1859524 | 0.43 | 7 | 46,813,415 | 4.4X10^-2^ | **6.9X10^-5^(16)** |  | |
| platelet aggregation (ADP-induced) | rs1038325 | 0.42 | 11 | 23,199,413 | 4.0X10^-4^ | **1.7X10^-5^(6)** |  | |
| platelet aggregation (collagen-induced) | rs1940484 | 0.32 | 11 | 98,447,082 | 3.0X10^-2^ | **9.0X10^-6^(4)** | *CNTN5* | |
| platelet aggregation (collagen-induced) | rs718617 | 0.25 | 13 | 26,865,841 | 5.9X10^-3^ | **2.7X10^-5^(10)** | *MTIF3* | |
| platelet aggregation (collagen-induced) | rs959898 | 0.41 | 13 | 92,534,850 | 1.6X10^-2^ | **9.3X10^-5^(25)** |  | |
| platelet aggregation (Epi-induced) | rs1456017 | 0.43 | 2 | 46,819,278 | 8.2X10^-3^ | **5.0X10^-6^(2)** | *CRIPT;AK124249;*  *AK127488;SOCS5* | |
| platelet aggregation (Epi-induced) | rs2353307 | 0.23 | 2 | 51,299,142 | 1.5X10^-1^ | **8.1X10^-5^(21)** |  | |
| platelet aggregation (Epi-induced) | rs764533 | 0.40 | 7 | 22,927,115 | 1.6X10^-2^ | **4.0X10^-6^(1)** | *BC009555;AF111113;KLHL7;*  *BC039585* | |
| platelet aggregation (Epi-induced) | rs2722292 | 0.24 | 7 | 37,597,821 | 2.8X10^-2^ | **4.0X10^-5^(13)** | *GPR141* | |
| platelet aggregation (Epi-induced) | rs1562871 | 0.17 | 8 | 128,470,954 | 2.6X10^-2^ | **2.7X10^-5^(11)** | *AF268618* | |
| RBCC | rs9253 | 0.18 | 1 | 37,627,906 | 4.2X10^-6^ | **1.1X10^-5^(5)** | *FLJ11730;BC016328* | |
| RBCC | rs10517484 | 0.46 | 4 | 60,531,780 | 1.3X10^-3^ | **7.7X10^-5^(19)** |  | |
| RBCC | rs727979 | 0.14 | 6 | 149,635,613 | 7.5X10^-6^ | **2.6X10^-5^(9)** | *MAP3K7IP2* | |
| RBCC | rs10484952 | 0.38 | 6 | 159,852,743 | 1.7X10^-1^ | **6.4X10^-5^(15)** |  | |
| RBCC | rs1965861 | 0.33 | 6 | 159,853,132 | 1.2X10^-1^ | **9.0X10^-5^(23)** |  | |
| Viscosity | rs10484893 | 0.13 | 6 | 24,996,530 | 4.7X10^-2^ | **3.4X10^-5^(12)** | *AB002384* | |
| Viscosity | rs2739122 | 0.41 | 8 | 134,247,063 | 4.6X10^-2^ | **5.0X10^-6^(3)** | *TG;AY196486;WISP1;*  *AY196487* | |
| ^*^ For Hgb, MCH and RBCC reported here, multivariable adjusted residuals from average of measurements over exam cycles 1 and 2 were used; for all platelet aggregation phenotypes, and viscosity reported here, multivariable adjusted residuals from measurements at examination cycle 5 were used.  ^†^ Physical position is in base pair (bp) and based on the May 2004 human reference sequence (NCBI Build 35).  ^††^ P-value from GEE genotype association test. | | | | | | | |  |
| ^†††^ P-value from family-based association test using the FBAT program, and rank of FBAT p-values in ascending order. | | | | | | | |  |
